# Relationship between Phylogeny and Immunity Suggests Older Caribbean Coral Lineages Are More Resistant to Disease

**DOI:** 10.1371/journal.pone.0104787

**Published:** 2014-08-18

**Authors:** Jorge H. Pinzón C., Joshuah Beach-Letendre, Ernesto Weil, Laura D. Mydlarz

**Affiliations:** 1 Department of Biology, University of Texas Arlington, Arlington, Texas, United States of America; 2 Department of Marine Sciences University of Puerto Rico, Mayagüez, Puerto Rico, United States of America; King Abdullah University of Science and Technology, Saudi Arabia

## Abstract

Diseases affect coral species fitness and contribute significantly to the deterioration of coral reefs. The increase in frequency and severity of disease outbreaks has made evaluating and determining coral resistance a priority. Phylogenetic patterns in immunity and disease can provide important insight to how corals may respond to current and future environmental and/or biologically induced diseases. The purpose of this study was to determine if immunity, number of diseases and disease prevalence show a phylogenetic signal among Caribbean corals. We characterized the constitutive levels of six distinct innate immune traits in 14 Caribbean coral species and tested for the presence of a phylogenetic signal on each trait. Results indicate that constitutive levels of some individual immune related processes (i.e. melanin concentration, peroxidase and inhibition of bacterial growth), as well as their combination show a phylogenetic signal. Additionally, both the number of diseases affecting each species and disease prevalence (as measures of disease burden) show a significant phylogenetic signal. The phylogenetic signal of immune related processes, combined with estimates of species divergence times, indicates that among the studied species, those belonging to older lineages tend to resist/fight infections better than more recently diverged coral lineages. This result, combined with the increasing stressful conditions on corals in the Caribbean, suggest that future reefs in the region will likely be dominated by older lineages while modern species may face local population declines and/or geographic extinction.

## Introduction

Immune defenses are critical for species success on ecological and evolutionary time scales [1]–[3]. As species diverge, new sets of genetic, biological and/or environmental conditions are encountered making it necessary for emerging species to trade off costs and benefits within and between traits [4], including those related to immunity [5]. Immunity plays an important role in the success of a given species and, in theory, evolves as species diverge [1], [6], likely conserving beneficial mechanisms from ancestral species [7]. Depending on selective pressures (e.g. resources and/or stressors), the immune system develops novel strategies and diversifies during speciation [6], [8], hence favoring individuals that survive pathogenic infections and other stressful events [7], [9]. In closely related species, the study of immunity in relation with phylogeny can provide insight into the selective forces at work and the way organisms and populations may respond to them [6], [10], [11].

The deterioration of coral reef ecosystems has been associated, among other factors, with changes in environmental conditions (e.g. increased water temperature and ocean acidification) and a significant increase in number of coral diseases and epizootics [12]–[16]. However, the evolutionary importance of immune traits in corals has not yet been evaluated. In other organisms such as fleas [17], termites [18] birds [19], [20], and vertebrates in general [21], immune traits are related to phylogeny. In corals, a recent taxonomic reorganization [22] facilitates assessment of trait variation and their relationship to life-history.

Scleractinia, the Cnidarian Order grouping all reef-building corals, is divided into two divergent groups termed Robust and Complex corals [23]–[26], each composed of several non-monophyletic families [27] with different evolutionary histories between the Atlantic and the Indo-Pacific regions [28]. Modern Caribbean scleractinians are grouped in at least six families, representing both Robust and Complex corals [27]. Recently, the Caribbean has become a disease ‘hot spot’ due to the high number of coral diseases, disease outbreaks and their widespread geographical range [16], [29], [30].

The innate immune system in corals is comprised of conserved components similar to those of other invertebrate [31], [32] and vertebrate species [33], [34] including the three general phases in the response to infection: recognition, signaling and effector responses [35]. While recognition receptors and several signaling pathways (e.g. toll and complement pathways) are activated upon pathogen recognition, many immune components, such as some effector mechanisms, show constitutive activity (i.e. non-pathogen induced or basal levels). Some of the better studied immune mechanisms in corals include the melanin synthesis cascade (e.g. prophenoloxidase and phenoloxidase) [31], [35], [36], antimicrobial compounds [31], [36]–[38], and antioxidants (e.g. superoxide dismutase, peroxidase and catalase) [31], [32], [35], [36]. Combined, these immune components provide corals with the capability to control the presence and combat proliferation of pathogens [31], repair tissue [39], and reduce levels of reactive oxygen species generated during infections and associated stress [40], [41]. Constitutive levels of prophenoloxidase, melanin [35] and antimicrobial activity [42] have been linked to disease resistance [32], [35], [43]–[45] suggesting an active investment in components of the immune system [10].

Investment in immunity has been related to different life history traits [1]. Since all biological traits, including those involved in immunity, tend to vary within and across populations and/or species during speciation, different evolutionary pressures (e.g. new pathogens or changes in climate and/or local environmental factors) can potentially result in distinct, species-specific, immune defenses. In corals, analyses of ecological strategies suggests related groups are affected similarly by environmental stressors and diseases [16], [46]–[48], but little is known about the role innate immunity plays in this pattern. Phylogenetic signals of biological traits are expected to be common across different groups of organisms [11], [49], but it is unknown what types of traits or what traits themselves will show a signal. Detecting a phylogenetic signal in coral immunity can provide insight into the evolution of the coral' immune system, and help explain the current pattern of disease resistance and its implications for the future of coral species and coral reefs, more so in light of global climate change and increased disease pressure [11], [50], [51].

In this study, we tested for the presence of a phylogenetic signal in immune traits and epizootics in Caribbean corals. We characterized the constitutive levels of six distinct immune traits in 14 of the most common and widely distributed coral species in the wider Caribbean. This represents ∼20% of the total diversity of scleractinian corals in the region [52]. We also compiled published disease data and determined the levels of immunocompetence of the studied species into two different metrics: Number of diseases (including both tissue loss diseases and growth anomalies) affecting each species, and mean prevalence, or the proportion of infected individuals in the population of a given species. These data sets were incorporated with the phylogeny of the host species (based on the 28S rDNA region) into three phylogenetic signal estimators (Bloomberg's K, Moran's I and Abouheif test).

The null hypothesis was that in scleractinian corals there is no relationship between phylogeny and levels of various enzymes involved in constitutive immunity and no correlation with disease parameters. Our results show co-variation between constitutive immunity and phylogeny in Caribbean corals, with species in older lineages (lineage defined as extant species and their ancestors) grouping together with lower number of disease and disease prevalence, than the modern lineages. Combined with other life history traits, these older lineages may be better suited to survive current and emerging diseases in the wider Caribbean.

## Materials and Methods

### Sample collection

For this study, 14 of the most common Caribbean scleractinian species comprising10 genera and 6 families [as defined by: 22, 27, 28, 53], were collected from several reefs (Media Luna - 17° 56.096 N; 67° 02.911 W, Turromote - 17° 56.097 N; 67° 01.130 W, Conserva - 17° 57.831 N; 67° 02.940 W, Pinnacles - 17° 55.963 N; 67° 00.714 W, Corral - 17° 56.986 N; 67° 00.504 W and Isla Cueva - 17° 57.599 N; 67° 04.827 W) off La Parguera, southwest coast of Puerto Rico (Table 1). To prevent seasonal or environmental effects, all samples were collected during the summer (northern hemisphere), the second week of August 2012. This collection represents approximately ∼20% of the total number of scleractinian coral species in the region [54]. Many of these species, i.e. *Montastraea cavernosa, Orbicella* spp. ( =  *Montastraea annularis* complex), *Diploria, Pseudodiploria*, and *Porites* spp. are common and widely distributed through the region. Other groups, such as acroporids (*A. palmata, A. cervicornis* and *A. prolifera*) and pocilloporids (e.g. *Madracis* spp.) were not collected due to strict limits on sampling and manipulation.

**Table 1:** List of scleractinian coral species used to measure constitutive immunity and its variation across taxonomic levels.

Family	Genus	Species	NCBI accession
Poritidae (III)	*Porites*	*Porites astreoides*	EU262830
Poritidae (III)	*Porites*	*Porites porites*	EU262878
Siderastreidae (IX)	*Siderastrea*	Siderastrea radians*	KJ946356
Meandrinidae (XII)	*Dendrogyra*	*Dendrogyra cylindrus*	EU262819
Meandrinidae (XII)	*Meandrina*	Meandrina jacksoni*	KJ946355
Meandrinidae (XII)	*Meandrina*	*Meandrina meandrites*	EU262815
Montastraeidae (XVI)	*Montastraea*	*Montastraea cavernosa*	EU262810
Merulinidae (XVII)	*Orbicella*	*Orbicella annularis*	HQ203479
Merulinidae (XVII)	*Orbicella*	*Orbicella franksi*	EU262849
Merulinidae (XVII)	*Orbicella*	*Orbicella faveolata*	EU262781
Mussidae (XXI)	*Diploria*	*Diploria labyrinthiformis*	EU262772
Mussidae (XXI)	*Pseudodiploria*	Pseudodiploria strigosa*	KJ946354
Mussidae (XXI)	*Mussa*	*Mussa angulosa*	EU262869
Mussidae (XXI)	*Mycetophyllia*	*Mycetophyllia aliciae*	EU262809

Underlined groups within the family and genus categories represent the sequence used for that group in the phylogenetic signal assessments. Accession numbers correspond to the sequence of the 28S rDNA region used for each species. (* =  Species sequenced in this project).

Small fragments from a total of 140 apparently healthy (i.e. with no signs of disease or bleaching) colonies (10 per species) were sampled. All samples were collected under the specification of research collection permits to the Department of Marine Science University of Puerto Rico – Mayagüez (UPRM), issued by the Department of Natural Resources of Puerto Rico. A fragment of approximately 5 cm^2^ was carefully removed from the top of each massive/crustose colony with a hammer and a chisel. Small branches were broken from branching colonies. For the “free-living” *Siderastrea radians*, rolling stones larger than 4 cm in diameter were collected in shallow sea-grass beds next to the reefs. All samples were stored in individually labeled sterile Whirl-pack bags (Fisher Scientific, Waltham, MA), transported in seawater to the laboratory and flash-frozen in liquid nitrogen. Frozen samples were stored at −80°C, shipped to the University of Texas at Arlington (UTA) in dry ice and kept at −80°C until further analyses.

### DNA extractions and PCR amplifications

The NCBI database has a significant number of sequences from most of the corals used in this project. The 28S rDNA region has been sequenced (as of September 2013) for 11 of the 14 species in this study (Table 1), and phylogenetic reconstructions showing similar topologies to other molecular markers, and divergence time estimation using fossils (*Caryophyllia* spp., *Flabellum* spp. and Dendrophyllidae), were available. Sequences for three species (*Meandrina jacksoni* GenBank KJ946355, *Pseudodiploria strigosa* KJ946354 and *Siderastrea radians* KJ946356) were generated in this project after extraction of DNA using a modified protocol from LaJeunesse *et al*. [55]. A small fragment (∼3 mg) of skeleton and tissue was mixed with (of glass beads (∼200 µl, 1 mm, Ceroglass, Columbia, TN) and 600 µl of a cell lysis solution (0.2 M Tris, 2 mM EDTA, 0.7% SDS, pH 7.6) and shaken on a BioSpec (Bartlesville, OK) beadbeater for 100 seconds. Proteinase K (3 µl −20 mg/ml) was added and incubated at 65°C for 1 hour. The incubation was followed by protein precipitation with ammonium acetate (250 µl −9 M) and freezing at −20°C. The frozen extract was centrifuged (10,000 G for 15 minutes) and the supernatant removed, mixed with 600 µl of isopropanol (100%) and centrifuged (10,000 G for 5 minutes). The DNA pellet was washed with 70% ethanol, air dried, and resuspended in 75 µl of distilled water and stored at −20°C.

The 28S rDNA region was amplified using the 28SROM.IFw (5′-GGCGACCCGCTGAATTCAAGCATAT-3′) and 28SDES.VRv 5′-GGTCTTTCGCCCCTATACTC-3′) primers [56]. Reactions were performed using Perfect Taq Plus DNA Polymerase (5-Prime, Gaithersburg, MD) following the manufacturer recommended reaction composition on 2 µl of 1∶40 dilutions of the extracted DNA (final reaction volume 25 µl). Amplifications consisted of 35 cycles of 95°C, 52°C and 72°C steps, each for 30 seconds. Amplified products were cleaned with ExoSap (Affymetrix, Santa Clara, CA) and sequenced with the forward primer using Big Dye 3.1 terminator mix (Applied Biosystems/Life Technologies, Grand Island, NY) on an ABI Hitachi 3730XL genetic analyzer at UTA' Genomics Core Facility. DNA sequence chromatograms were reviewed and edited using Geneious Pro 5.0 [57]. The resulting sequences were combined with those obtained from the NCBI data base (657 to 685 bp) and alignments were performed on ClustalW using a gap-opening penalty of 15 and a extension penalty of 6 [58]. Phylogenies were constructed on MrBayes [59], using a general time-reversible model with gamma distributed rate heterogeneity (GTR+G) as substitution model, a chain length of 1,100,000 and 100,000 burn-in (phylogenies can be found in Text S1).

In order to determine the approximate age of the studied lineages, that is the extant species and their ancestors, we calculated the divergence times on the 28S rDNA based phylogeny (Text S2). Divergence times were determined with BEAST 1.7.5 [60], using a relaxed-clock uncorrelated lognormal allowing for nucleotide substitutions rates to vary between lineages [26]. The tree prior used a Yule process and the model of substitution was set to gamma distributed rate heterogeneity as suggested by jModeltest 2.1.3 [61], with invariant sites (GTR+G+I). Node ages and posterior probabilities were estimated on a run with 10,000,000 generations and saving the topologies and parameters every 1,000 generations. In order to estimate the early divergence of the studied groups, calibrations were done using Dendrophyllidae (∼127 Mya; including sequences of *Tubastrea coccinea, Cladopsamia gracilis, Leptosammia pruvoti, Endopachys grati, Enollopsamia rostrata* and *Balanophyllia* spp.), *Caryophyllia* spp. (∼160 Mya) and *Flabellum* spp. (∼77.5 Mya) as suggested by Stolarski *et al*. [26]. Phylogenetic reconstruction within BEAST was performed using Mean heights and node heights, a prior probability of 0.1 and 1,000,000 burn-in.

### Protein extractions and immune assays

Protein extractions and enzymatic assays followed protocols previously used to study coral immunity [31], [32], [35], [36], [43]. Coral tissue was airbrushed from the skeleton using a Paasche single action artistic airbrush (Paasche Airbrush Company, Chicago, IL) with minimal amounts (5–6 ml) of Tris buffer (100 mM Tris, pH 7.8+0.5 mM dithiothreitol). To break open cells and extract proteins, tissue slurries were homogenized using a tissue homogenizer (Powergen 125, Fisher Scientific, Waltham, MA) for 1 minute on ice. One ml of the tissue slurry was added to pre-weighted 1.5 ml microfuge tubes for melanin concentration estimates. All homogenates were centrifuged at 90×G for 10 minutes and the supernatant was recovered. Protein concentrations were estimated using the RED_660_ protein assay (G Biosciences, Saint Louis, MO) and standardized to a standard curve of bovine serum albumin.

We performed assays for six immune traits: prophenoloxidase, melanin concentration, superoxide dismutase, peroxidase, catalase activity and inhibition of bacterial growth. Prophenoloxidase was tested on 20 µl of the extract, mixed with 20 µl of sodium phosphate buffer (50 mM, pH 7.0) and 25 µl of Trypsin (0.1 mg/ml). The reaction was initiated by adding 30 µl of dopamine (10 mM) as a substrate. Change in absorbance was measured every 30 seconds at 490 nm for 15 minutes and activity calculated during the linear range of the curve (1–5 minutes).

Melanin concentration was assessed on the melanin-reserved portion of initial tissue slurry after freeze-drying (VirTis BTK, SP Scientific, Warminster, PA) for 24 hours. The resulting dried tissue was weighed and the melanin extracted with 400 µl NaOH (10 M). Extraction was done at room temperature for 48 hours at which time the tissue particles were centrifuged (90×G) for 10 minutes. 60 µl of the supernatant were used to determine the absorbance at 410 nm. Resulting values were standardized to a dose-response curve of commercial melanin (Sigma-Aldrich, Saint Louis, MO).

Superoxide dismutase activity was determined with the SOD Determination kit (#19160, Sigma-Aldrich, Saint Louis, MO) following manufacturer's instructions. Absorbance at 450 nm was measure in wells containing coral protein extracts and superoxide dismutase controls and compared to untreated samples. The inhibition was normalized by mg protein and presented as superoxide dismutase activity units per mg protein.

Peroxidase activity was assessed on 10 µl of extract with 40 µl phosphate buffer (0.01 mM, pH 6.0) and 25 µl Guaiacol (25 mM). Activity was monitored for 15 minutes, recording the absorbance at 470 nm every 30 seconds. Peroxidase is presented as change in absorbance per mg protein per minute.

Catalase was measured as the change in hydrogen peroxide concentration after mixing 5 µl of the protein extract with 45 µl of sodium phosphate buffer (50 mM, pH 7.0) and 75 µl of 25 mM H_2_O_2_. Samples were loaded on UV transparent plates (Grainer Bio-one, Monroe, NC) and read at 240 nm every 30 seconds for 15 minutes. Catalase activity was estimated as change in hydrogen peroxide concentration per mg of protein during the first minutes of the reaction.

The percent inhibition of bacterial growth was assessed against *Vibrio alginolyticus* (Strain provided by K. Ritchie, Mote Marine Laboratory, GenBank # X744690). This particular bacterial strain was isolated from *Orbicella faveolata*, and has been implicated in Yellow Band Disease [62], [63], and thus offers a good estimate of antibiotic activity in a broad range of corals. Bacteria were grown in salt amended (2.5% NaCl) Luria Broth (EMD Chemicals, Gibbson, NJ) for 24 hours prior to use in the assay. The resulting bacteria culture was diluted to a final optical density of 0.2 at 600 nm. 140 µl of the culture suspension was added to each well along with 60 µl of the coral extract. To detect possible effects of the media and the airbrushing buffer, controls with 60 µl of Tris Buffer (100 mM Tris, pH 7.8+0.5 mM dithiothreitol) were included on each experimental plate. Plates were incubated in the spectrophotometer for 6 hours at 29°C, determining the absorbance at 600 nm every 10 minutes. The change in absorbance during the logarithmic phase were used to determine the growth rate of each samples and the proportion of this rate to that of the bacterial control provided the percent inhibition for each samples.

These immune assays are ideal for comparative immunity studies because they are not species-specific [10]. All assays were conducted on a Synergy 2 Microplate Reader (Biotek Instruments, Winooski, VT) and standardized to mg of protein when applicable. Constitutive levels of all six immune traits were compared between older and modern coral lineages (groups described in the results section). Comparisons were assessed with a t-test assuming unequal variances across traits.

### Data analysis and phylogenetic signal estimations

In order to test the hypothesis that closely related taxa have similar activity of constitutive immune components, the data was partitioned in three groups corresponding to each of the clades of interest: family, genus and species (Table S1). Results from the immune assays were averaged for each taxon on each level. Additionally, to obtain an integrated measure of immunity the first component scores from a principal component analysis (PC1) with all individual immune measures was used as an additional category. Principal components reduce dimensions and convert multiple variables into composite indicators [64], improving the analysis of immune capacity in relation to other biological/environmental parameters [65]. The analyses were done in JMP 10.0.0 (SAS Institute, Cary, NC).

In order to detect a phylogenetic signal, the immune data sets were compared against family, genera and species phylogenetic reconstructions (Text S1). The genus and family phylogenies were built with subsets of selected sequences from each group (underlined categories in Table 1). For example, *Pseudodiplora strigosa* 28S sequence was used as the representative for the *Pseudodiplora* genus.

The possibility of phylogenetic signals in coral constitutive immune levels was tested with Bloomberg's K [49] and Moran's I measures as described by Gittleman and Kot [66] and by Abouheif test [67]. K assumes the data follows a Brownian motion model (BM) and compares the observed phylogenetic signal with that of the trait under the BM model. Higher K values for a particular trait represent a stronger phylogenetic signal, and zero values indicate no effect of phylogeny [17], [49]. Moran's I (*I*) on the other hand, is a model-independent measure of autocorrelation in which the relation between the variation in the trait and the phylogenetic distance is established. In this method, the data is divided into the phylogenetic component and the trait component and correlarograms are built to determine the effect of ranks and distances [66]. Lastly, the Abouheif [67] test (*A*), modifies Moran's I to successfully detect phylogenetic signal of different traits on phylogenies with both low and high number branches. All phylogenetic signal tests were performed on R using geiger, carper, picante, adephylo and phylobase packages.

A Spearman's rank order correlation index was used to determine correlations between each immune measure and estimates of number of diseases and prevalence (proportion of infected individuals in the population of a given species at a given time) for each taxonomic level (i.e. species, genus and family). Disease parameters (number of diseases and prevalence) were compiled from literature reporting epizootic events in the Caribbean from 1997 to 2005. Only manuscripts with species level resolution, and infected or diseased colonies specified as a subset of the total population surveyed were used so that number of diseases and prevalence estimates could be normalized across reports. Prevalence was estimated from the reports by combining data from all surveyed diseases (e.g. Black Band, White Band, White Plague, Yellow Band, Dark Spots, and growth anomalies) for each species. A total of 12 reports with appropriate information were found from different locations in the Caribbean, including Bermuda, Florida, Bahamas, Puerto Rico, Saint Croix, Bonaire, Yucatan-Mexico, Colombia and Venezuela (Table 2) [29], [30], [68]–[77]. The geographic coverage and extent of the disease surveys in these reports provided a very robust data set for both number of diseases and disease prevalence, thus truly representing the disease dynamics across the region. Phylogenetic signal in the number of diseases and prevalence was estimated as described above.

**Table 2:** List of references, reports and reviews of coral disease parameters from various locations in the Caribbean used to obtain data for the 14 species used in the phylogenetic signal estimates.

Reference	Location	Survey year(s)	Data used
Sutherland *et al.*, 2004	Caribbean Wide	Review	Number of diseases
Weil, 2004	Caribbean Wide	Review of 1999–2002	Number of diseases and prevalence
Kaczmarski *et al.*, 2005	Saint Croix	2001	Prevalence
Voss and Richardson, 2006	Lee Stocking Island - Bahamas	2002, 2003	Prevalence
Santavi *et al.*, 2001	Florida	1997, 1998	Number of diseases
Ward *et al.*, 2006	Yucatan Peninsula - Mexico	2004	Prevalence
Garzón-Ferreira *et al.*, 2001	Colombia	1998	Number of diseases
Navas-Camacho *et al.*, 2010	Colombia	1998–2004	Number of diseases
Gil-Agudelo *et al.*, 2010	Colombia	1998–2005	Number of diseases
García *et al.*, 2003	Los Roques - Venezuela	1999	Number of diseases and prevalence
Croquer *et al.*, 2003	Los Roques - Venezuela	2000	Number of diseases and prevalence
Croquer *et al.*, 2005	Los Roques - Venezuela	2000, 2001	Prevalence

Data used included the number of diseases affecting each species and average disease prevalence (proportion of infected individuals on a population of a given species) for all diseases affecting each species.

## Results

### Phylogenetic reconstruction and time of divergence

**Figure 1:**
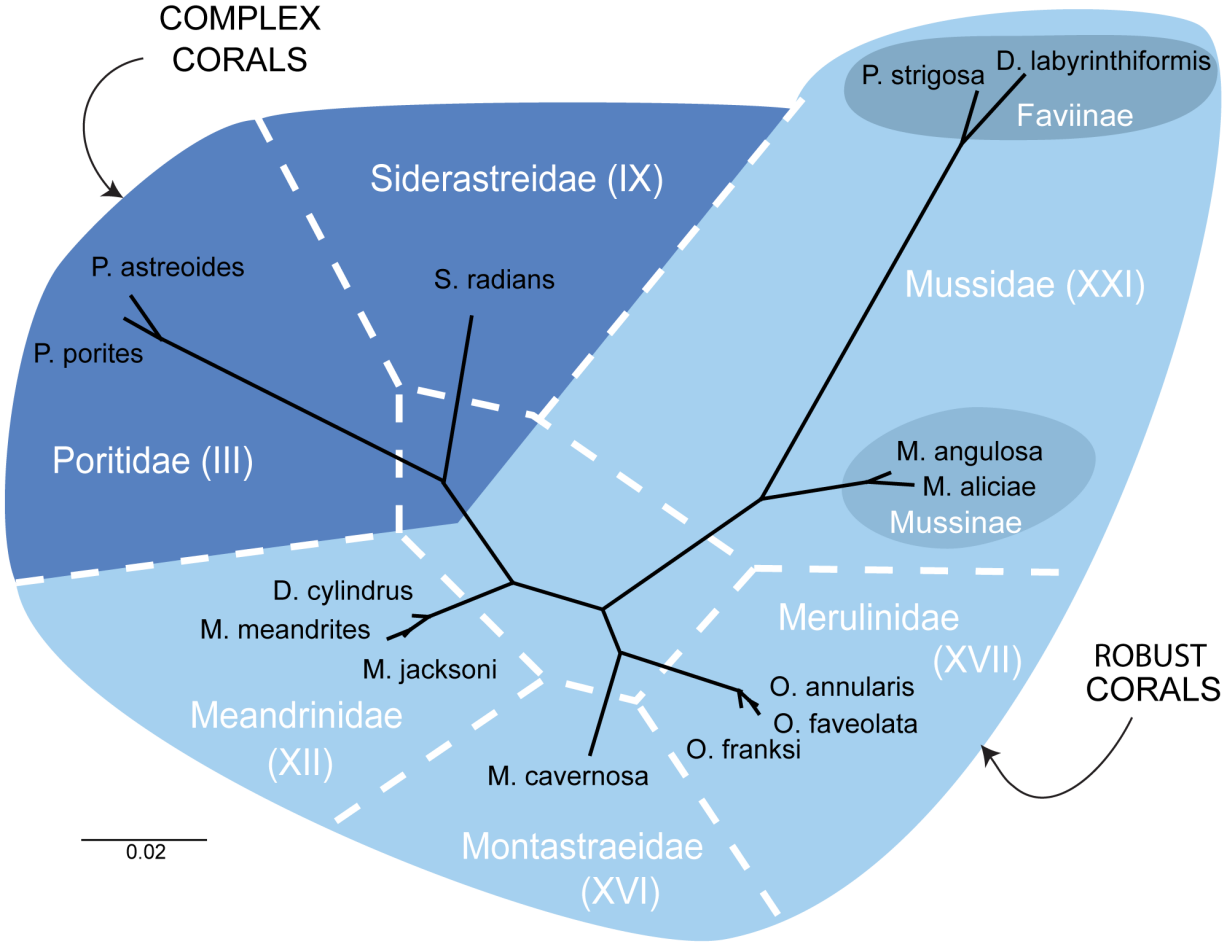
Un-rooted Bayesian phylogenetic reconstruction (dark line) using the 28S rDNA (657–685 bp) region from 14 species of Caribbean corals used in this study. The dotted lines represent the family groupings as recently proposed by Budd et al. [22]. All nodes in this tree have support (posterior probability) values higher than 0.80.

Sequences of the 28S rDNA region for three corals were generated and in each case these newly sequenced species clustered according to current taxonomy; *Meandrina jacksoni* with its sister species *M. meandrites, Pseudodiploria strigosa* with *Diploria labyrinthiformis* among the robust corals and *Siderastrea radians* with *Porites* spp. in the complex corals (Figure 1). The phylogenetic hypothesis obtained with the 28S rDNA region, using all 14 species, resolved the same groups delineated with other molecular markers and in other geographic locations [27], [53], [78].

**Figure 2:**
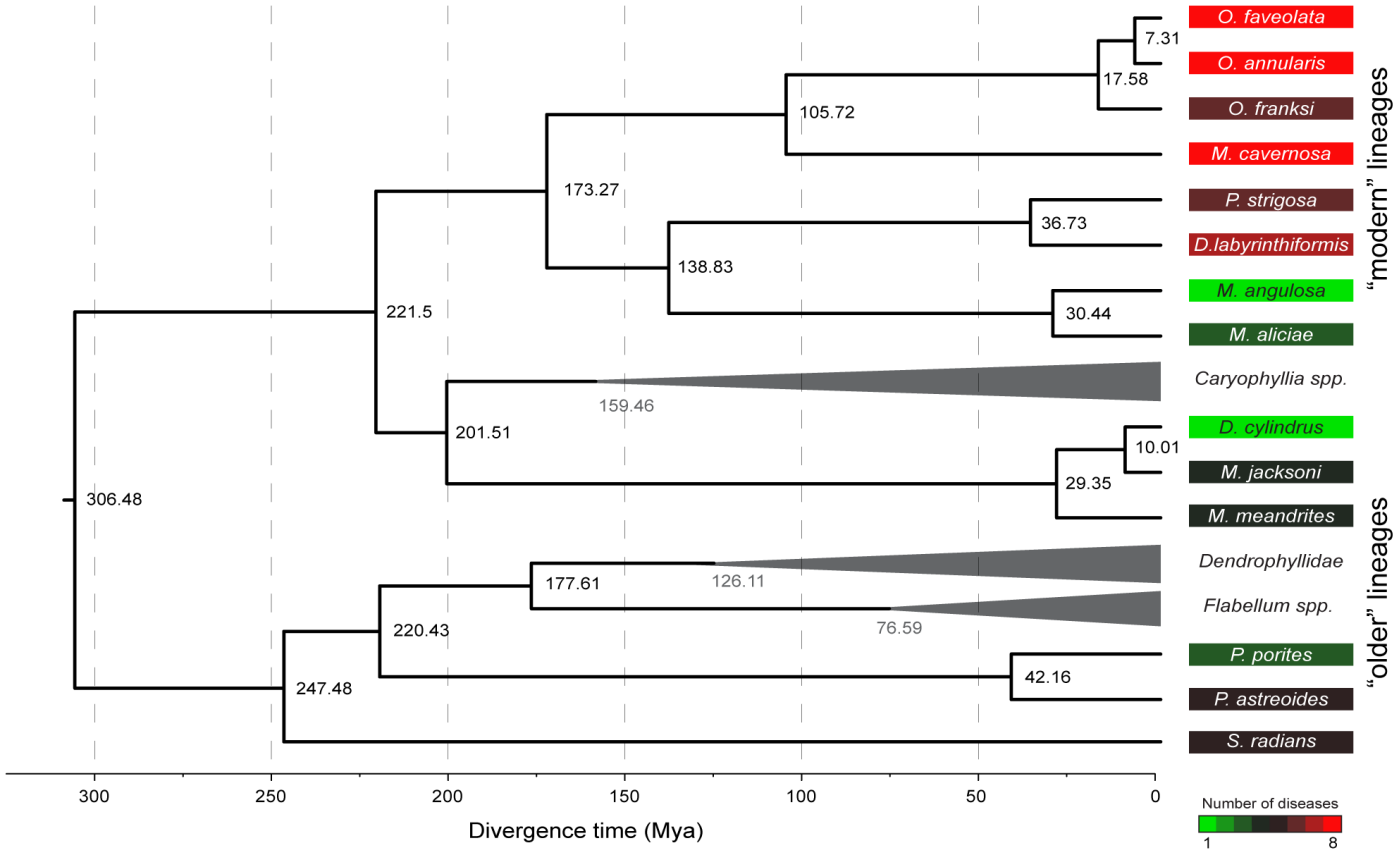
Phylogenetic reconstruction with the 28S rDNA region used to estimate the divergence time (dark values in the nodes) of the evolutionary lineages of 14 Caribbean coral species. Time of divergence was estimated with BEAST following the method by Stolarski et al. [26]. The molecular clock was calibrated with specimens from the family Dendrophyllidae at 127±3.5 Mya, and the genera *Caryophyllia* at 160±3.5 Mya and *Flabellum* at 77.5±3.5 Mya, represented by gray triangles and values. Number of diseases affecting each species is shown in the colored squares. Disease data was compiled from the literature (Table 2) and corresponds to surveys between 1997 and 2005 throughout the Caribbean.

Divergence times of the included lineages (i.e. extant species and ancestors) derived from the molecular clock show that *Siderastrea radians* (∼247 Mya) and *Porites* spp. (∼220 Mya) diverged early during the Triassic and appear as ancestral lineages among the Complex corals. Among the robust corals, the family Meandrinidae also showed early divergence (∼201 Mya), followed by a recent radiation into the current species (∼30–42 Mya; Figure 2). The other Complex lineages evolved within the last ∼100 to 150 Mya (Figure 2). Faviinae (*D. labyrinthiformis* and *P. strigosa*) and Mussinae (*Mycetophyllia aliciae* and *Mussa angulosa*) diverged ∼138 Mya, with current species diverging as recently as 40 Mya. The genera *Orbicella* and *Montastraea* originated ∼105 Mya, with *Orbicella* radiating between ∼17 and ∼7 Mya (Figures 1 and 2). This allowed the sampled lineages to be grouped into older (*S. radians, P. porites, P. astreoides, D. cylindrus, M. meandrites* and *M. jacksoni*) and modern (*M. aliciae, M. angulosa, D. labyrinthiformis, P. strigosa, M. cavernosa, O. faveolata, O. annularis* and *O. franksi*) lineages.

### Phylogenetic signal in coral immunity and disease

Results from the three phylogenetic signal measures were similar across taxonomic levels (i.e. species, genus and family). These analyses revealed different patterns of phylogenetic signal for each independent immune measure (Table 3). Significant correlations between phylogeny and immune traits were found in melanin concentration at the species level (Moran's I - *I*_sp_ = 0.105, p = 0.036), peroxidase activity at the genus (*I*_genus_ = 0.451, p = 0.006; *A*_genus_ = 0.524, p = 0.005) and species levels (Bloomberg's K - K_sp_ = 1.269, p = 0.001; *I*_sp_ = 0.488, p = 0.002; *A*_sp_ = 0.583, p = 0.002) and percent inhibition of bacterial growth at the family level (K_family_ = 1.637, p = 0.010, *I*_family_ = 0.082, p = 0.048). Variation in prophenoloxidase, superoxide dismutase and catalase activities did not show a significant phylogenetic signal at any level (Table 3).

**Table 3:** Bloomberg's *K*, Moran's *I* and Abouheif I test (*A*) values, testing for phylogenetic signal in constitutive immunity (six individual measures and the integrated measure - PC1) and number of diseases affecting each species and prevalence (proportion of individuals infected in the population of a given species) at three taxonomic levels (family, genus and species) within 14 scleractinian coral species from the Caribbean Sea (bolded values show significant phylogenetic signal).

Individual immune measures							Integrated Measure	Disease Parameters	
Melanin Synthesis Pathway			Antioxidants			Antimicrobial Activity			
Prophenol oxidase		Melanin	Superoxide dismutase	Peroxidase	Catalase	% Inhibition of bacterial growth	PC1	Number of diseases	Prevalence
**Family**									
*K*	0.969	1.328	1.106	1.474	0.932	**1.637**	1.080	1.262	0.785
*I*	−0.018	−0.139	−0.171	0.218	−0.236	**0.082**	−0.130	0.153	**0.106**
*A*	0.116	**0.382**	0.101	0.326	0.030	0.266	0.122	0.248	0.182
**Genus**									
*K*	0.217	0.874	0.890	0.903	0.407	0.848	0.962	0.923	0.460
*I*	−0.067	−0.156	0.067	**0.451**	−0.032	0.187	0.219	0.226	−0.036
*A*	0.039	−0.062	0.247	**0.524**	0.008	0.252	**0.325**	0.358	0.011
**Species**									
*K*	0.252	0.168	0.534	**1.269**	0.329	0.321	0.504	**0.747**	**1.102**
*I*	−0.110	**0.105**	0.188	**0.488**	−0.020	0.182	**0.251**	**0.292**	**0.370**
*A*	−0.041	0.114	0.213	**0.583**	0.005	0.202	**0.271**	**0.351**	**0.544**

Principal Component Analysis revealed that Principal Component 1 (PC1) explained 33.5% of the variation, Principal Component 2 (PC2) 25.1% and Principal Component 3 (PC3) 23.3%. PC1 values were similar between closely related species showing a significant phylogenetic signal at the genus (*A*_genus_ = 0.325, p = 0.037) and species (*I*_sp_ = 0.251, p = 0.036; *A*_sp_ = 0.271, p = 0.049; Table 3) levels. PC2 and PC3 did not show a significant phylogenetic signal.

Closely related species have similar number of diseases and disease prevalence (proportion of individuals infected in the population of a given species). The number of diseases showed significant phylogenetic signal at the genus (*A*_genus_ = 0.358, p = 0.037) and species (*K*_sp_ = 0.747, p = 0.015, *I*_sp_ = 0.292, p = 0.026; *A*_sp_ = 0.351, p = 0.019) levels. Variation in disease prevalence was significant at the family (*I*_family_ = 0.106, p = 0.012) and species (K_sp_ = 1.102, p = 0.009; *I*_sp_ = 0.370, p = 0.004; *A*_sp_ = 0.544, p = 0.007) levels (Table 3).

### Correlations between disease parameters and immune measures

**Figure 3:**
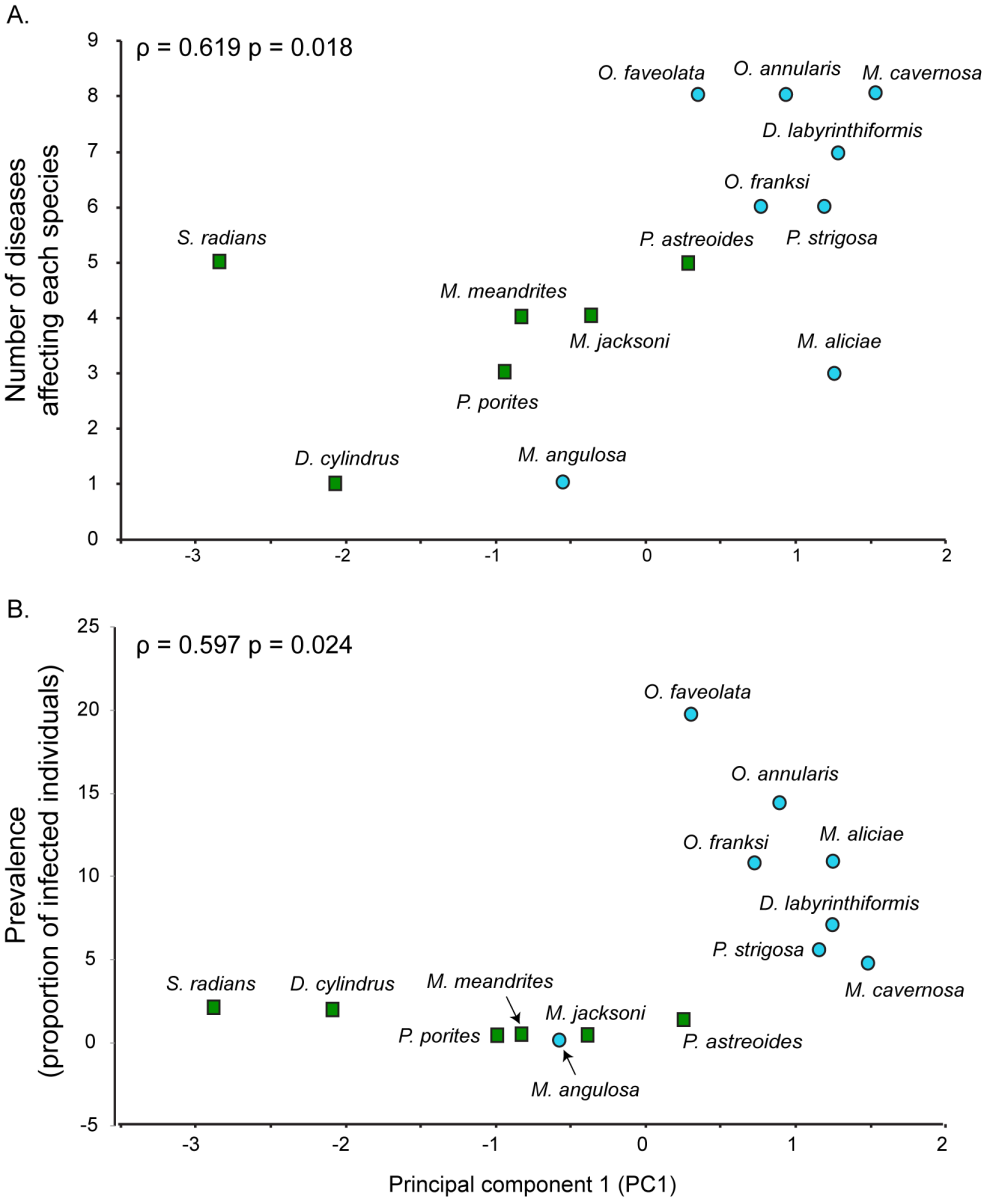
Correlation between (A) number of diseases (number of diseases affecting each coral species) and (B) prevalence (proportion of individuals infected in the population of a given species) with constitutive levels of immunity as measured with the Principal Component 1 (PC1) obtained from Principal Component Analysis of six immune measures (prophenoloxidase, melanin concentration, superoxide dismutase, peroxidase, catalase and inhibition of bacterial growth). The correlations were determined with the Spearman's rank order index (ρ) for both disease susceptibility and prevalence. Green squares represent older, and blue circles modern lineages.

Several of the individual immune traits were significantly correlated to number of diseases and prevalence. Peroxidase (ρ = 0.971, p = 0.001) at the family level, prophenoloxidase (ρ = 0.667, p = 0.035), superoxide dismutase (ρ = 0.636, p = 0.048) at the genus level and prophenoloxidase (ρ = 0.564, p = 0.036) at the species level showed significant correlations with number of diseases. Only peroxidase (ρ = 0.971, p = 0.001) at the family level was correlated with disease prevalence (Table 4). The integrated measure of immunity (represented by PC1) was positively and significantly correlated with number of diseases (ρ = 0.619, p = 0.018) and prevalence (ρ = 0.597, p = 0.024; Figure 3) at the species level. Number of diseases increases as divergence time of the lineages decrease.

**Table 4:** Spearman's rank order correlations (ρ) between constitutive levels of six immune traits and number of diseases affecting each species and average prevalence (proportion of infected individuals on a population of a given species) of all diseases affecting each species, at three taxonomic levels (family, genus and species) among 14 scleractinian coral species from the Caribbean (significant correlations are bolded).

Individual immune traits							Integrated measure
Melanin Synthesis Pathway			Antioxidants			Antimicrobial Activity	
Prophenol oxidase		Melanin	Superoxide dismutase	Peroxidase	Catalase	% Inhibition of bacterial growth	PC1
**Number of diseases**							
Family	0.618	0.000	0.530	**0.971**	0.088	−0.795	0.706
Genus	**0.667**	0.073	**0.636**	0.422	0.428	−0.294	0.593
Species	**0.564**	−0.107	0.517	0.531	0.264	−0.340	**0.619**
**Disease prevalence**							
Family	0.618	0.000	0.530	**0.971**	0.088	−0.795	0.706
Genus	0.309	0.030	0.394	0.261	0.515	−0.491	0.588
Species	0.265	−0.128	0.358	0.484	0.230	−0.475	**0.597**

**Figure 4:**
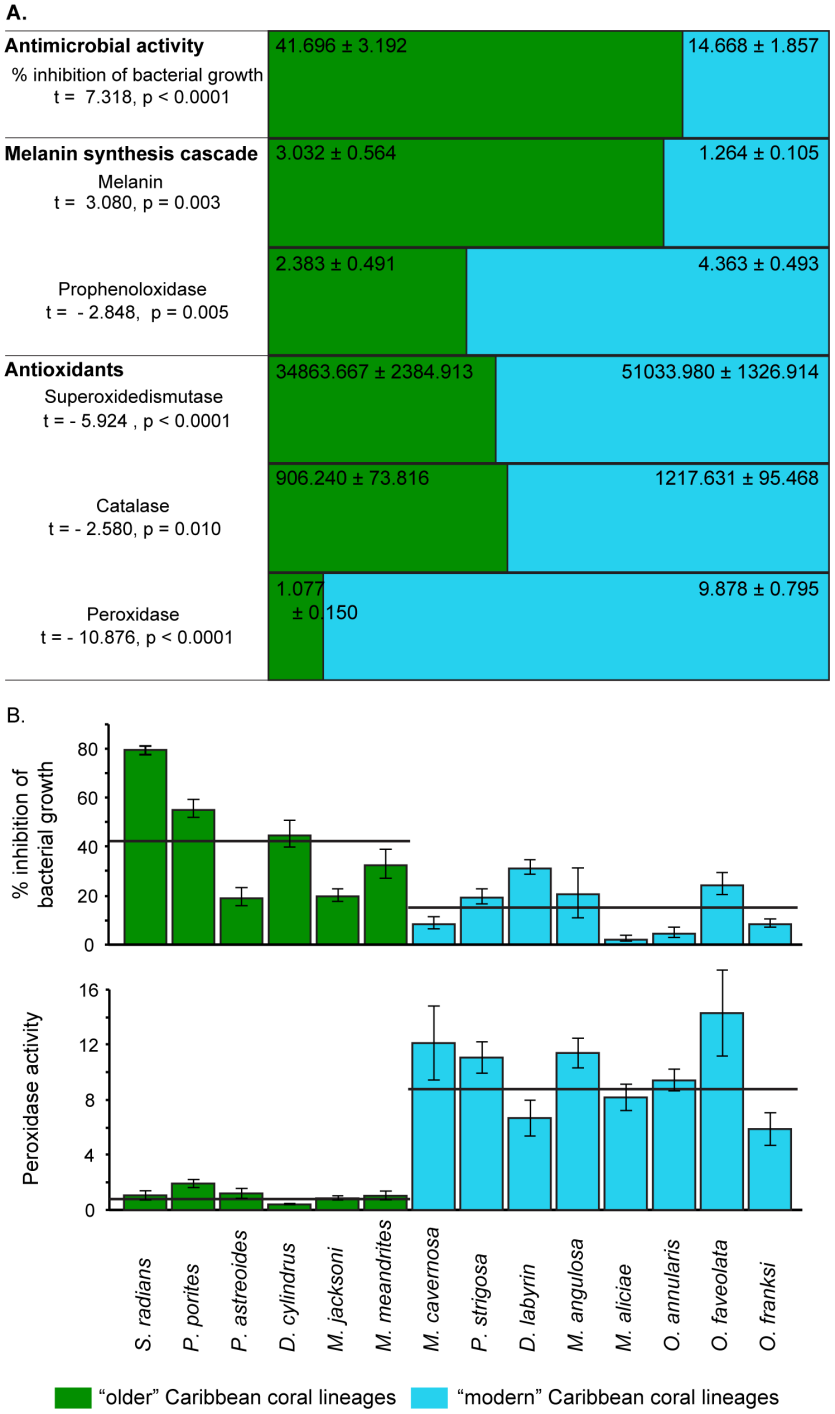
Proportion of the mean (± standard error) values of constitutive levels of six immune traits (percent inhibition of bacterial growth, melanin concentration, prophenoloxidase, superoxide dismutase, peroxidase and catalase activity) for older and modern Caribbean coral lineages (A). Pairwise comparisons for each trait using t-tests assuming unequal variances revealed significant differences between older and modern lineages for all traits. Older lineages showed higher mean values of melanin and percent inhibition of bacterial growth and lower values in the other traits compared to the modern lineages. Percent inhibition of bacterial growth and peroxidase activity (mean ± standard error of change in absorbance 490 nm per mg protein per minute) for 14 Caribbean coral species (B). Black lines represent the average immune assay values for each coral lineage.

Older lineages such as *Porites* spp. (known to be affected by at least 5 diseases), *Siderastrea radians* (5 diseases) and the Meandrinidae species (4 diseases) are affected by fewer diseases than modern groups such as *Diploria* (7 diseases), *Pseudodiploria* (6 diseases), *Montastraea* (8 diseases) and *Orbicella* (8 diseases; Figure 2) [29], [30], [68]–[75], [79]. Species from the subfamily Mussinae (*Mycetophyllia aliciae* and *Mussa angulosa*) were the exception with an average divergence time of ∼33 Mya and a low susceptibility (3 and 1 diseases respectively). The fact that *Mycetophyllia* and *Mussa* form a well-supported monophyletic group [22], [27], [78] that according to our molecular clock diverged ∼138 Mya (Figure 2), may explain this inconsistency. Pairwise (t-tests) comparisons showed all six immune traits levels are significantly different (p<0.050) between older and modern lineages (Figure 4).

## Discussion

Immune defenses are key to the evolutionary success of a species [3], but does the immune system retain ancestral characteristics, develop new or retain both ancestral and new defense strategies during speciation? [7] Answering this question in scleractinians is important since it can offer insight into the evolution of immunity and relevance to disease resistance and the future of corals in light of novel pathogens and climate change [50]. Results of this study indicate that the coral immune system, at least in Caribbean corals, has been molded by evolutionary history, with past selective pressures (biotic and abiotic) likely improving the innate immune system to respond more efficiently to new diseases [80]. Extant species, belonging to groups of species that have survived through more of these pressures, i.e. older coral lineages (*Porites* spp. ∼220 Mya, *Siderastrea radians* ∼247 Mya and *Meandrinidae* spp. ∼201 Mya), seem to be evolutionarily better equipped to cope/survive current conditions than more recent or modern lineages (*Orbicella* spp.  =  *Montastraea annularis* complex, ∼105 Mya).

Constitutive immunity, disease patterns and phylogenetic analyses of the 14 coral species in this study show some interesting patterns. Species from lineages that diverged more than 200 Mya are affected by fewer diseases, show lower disease prevalence, and have higher levels of some constitutive immune defenses. Some of these species, for example, *P. astreoides* and *S. radians* have ecological traits consistent with a resistant coral. Both are able to maintain normal physiological functions under stress [81], are brooders and exhibit a weedy-like dispersal and recruitment strategies [82]. Together with their high constitutive immune levels, these traits make for resistant and successful species [83] increasing survivorship and probably, species fitness.

Some of the modern lineages comprise another distinct group. Species in this group are affected by up to 8 different diseases, show high disease prevalence levels and have lower constitutive levels of some immune defenses. *Orbicella faveolata*, *O. annularis*, *Diploria labyrinthiformis*, and *Pseudodiploria strigosa* are among the species most heavily affected by disease in the Caribbean [16], [29], [30], leading to significant population losses in recent years.

This study did not include the Caribbean Acroporids (*Acropora palmata* and *A. cervicornis*). Acroporids in general are thought to have very low levels of immunity [35] and the Caribbean species were nearly extirpated over their geographic distribution by White Band Disease [84], [85]. High rates of clonal reproduction (i.e. low genetic variability) can result in higher population susceptibility to environmental and/or biological stressors [86]. Their clonal population structure [87] and life history traits (e.g. broadcasters with low recruitment success, fast growing branching morphology and high fragmentation rates, etc.) have rendered Caribbean acroporid populations highly susceptible to disease and contributed to their significant losses over their entire geographic distribution.

Overall, there was a relationship between constitutive levels of immune measures and diseases data that partitioned between old and modern lineages. Since the immune system is integrated, assessing several measures of immunity is ideal to gain a better understanding of both the potential to prevent and the capacity to fight infections [88], [89]. This study did not include induced immune responses or resistance to pathogens in experimental settings. However, it appears our multivariate immune measures are good indicators of disease susceptibility across these 14 species. Similar patterns to those detected here have been reported for Indo-Pacific corals, where specific markers of the melanin synthesis cascade were related to disease prevalence [35]. Our study integrates the melanin synthesis cascade, several antioxidants and general antimicrobial activity.

Among the specific immune traits, inhibition of bacterial growth and melanin concentration were higher in the older coral lineages, suggesting that these two mechanisms (among others not described here) may be important in conferring resistance. Prophenoloxidase and all the antioxidants were higher in the modern lineages. There is an inverse relationship between the melanin product and proteins in its biosynthetic cascade. Prophenoloxidase activity is likely to increase upon microbial stimulation, but corals with higher melanin concentrations may be able to prevent infection by deploying a melanin barrier before the need to engage into additional, more costly responses [90].

All antioxidants were more active in the modern lineages than in the older coral lineages. Antioxidants are an integral part of the stress response to both abiotic and biotic stressors. All modern lineages in this study are known to be susceptible to both elevated temperatures and to disease [31], [36], [43]. Increasing temperatures and/or pathogen load may keep these corals under a constant state of stress, thus elevating the levels of antioxidants at a cost to other defense pathways [32]. In addition to continuous stress, the algal symbiont type in each coral species may also play a part in oxidative stress that is reflected in the holobiont [91].

Variation in constitutive immunity among corals can also be the result of environmental and spatial gradients on a given reef. While our data are from one region and one time point, we fell they are a start to developing hypotheses to why older corals may be more resistant to diseases. In fact, we attempted to reduce the effects of the environment by collecting during the same season (within a week), and either sampling the same species from different reefs and different species from the same reef and from the same location of the colony. The exception was *Siderastrea radians*, which was collected in sea grass beds outside the reefs. However, *S. radians* immune measures clustered with those from both *Porites porites* and *P. astreoides*, supporting our hypothesis and minimizing the influence of the environment.

Associated organisms, *Symbiodinium* spp. and bacterial symbionts or communities may exert an important role in the immune defense of the coral host. Our protein extraction is enriched in host proteins but may include a minimal amount of *Symbiodinium* protein. The corals in this study associate with a variety of *Symbiodinium* species/strains (clades A, B, C, D). At least seven of the 14 coral species (e.g. *Pseudodiploria strigosa, Dendrogyra cylindrus, Meandrina meandrites, M. jacksoni, Orbicella faveolata, O. franksi* and *O. annularis*) are known to associate with *Symbiodinium* B1 [92]. The immune measures of these corals reflect the phylogeny of the coral host more than the presence of a particular symbiont.

Caribbean reefs are not likely to disappear, but rather change in community composition and structure [50], [93]. Coral species with advantageous life history traits will be more likely to dominate [94] with the immune capacity of individuals and/or populations serving as a critical piece of the puzzle. While our data are limited in the number of species and to the Caribbean region, the evidence presented here indicates that phylogenetically close scleractinians have comparable levels of constitutive immunity.

Our results support the ongoing observations that disease resistance varies considerably among coral taxa and provides a possible mechanistic basis for this variability. Caribbean species from older lineages seem better adapted to resist infections with high levels of melanin and antimicrobial activity and, as a result, may have experienced significantly lower mortality from disease outbreaks in the recent past [95]–[97]. The life history traits of many of these older lineages seem to make them well suited for survival under the current changing conditions. Correspondingly, older lineages of mammals appear less extinction-prone than previously predicted [98]. Recently diverged, modern Caribbean coral lineages on the other hand, have been severely affected by diseases and/or thermal stress with significant tissue loss and colony mortalities at local and regional geographic scales and some of these taxa could face local extinction in many areas [99].

## Supporting Information

Table S1 **Mean values (± standard error) of six immune traits (prophenoloxidase, melanin, superoxide dismutase, peroxidase, catalase and percent inhibition of bacterial growth), the integrated immune measure (PC1) and disease parameters (number of diseases and prevalence) used in the phylogenetic signal analyses on 14 Caribbean corals at three taxonomic levels (species, genus and family).**
(XLSX)

Text S1 **Phylogenetic reconstructions based on the 28S rDNA region of 14 Caribbean corals at three different taxonomic levels (species, genus and family).** These trees were used as input in the phylogenetic signal analyses.
(NEWICK)

Text S2 **Sequence alignment of the 28S rDNA region used to determine the divergence times of 14 Caribbean corals.** Dendrophyllidae (∼127 Mya; including sequences of *Tubastrea coccinea, Cladopsamia gracilis, Leptosammia pruvoti, Endopachys grati, Enollopsamia rostrata* and *Balanophyllia* spp.), *Caryophyllia* spp. (∼160 Mya) and *Flabellum* spp. (∼77.5 Mya) were used as calibration point in the molecular clock.
(NEX)
